# Community Based Participatory Research For The Development of a Compassionate Community: The Case of Getxo Zurekin

**DOI:** 10.5334/ijic.5707

**Published:** 2022-01-17

**Authors:** Naomi Hasson, Maider Urtaran-Laresgoiti, Roberto Nuño-Solinís, Itziar Moreno, Gorka Espiau, Maider Grajales, Janire Fonseca

**Affiliations:** 1 Fundacion Doble Sonrisa, ES; 2 Social Determinants of Health and Demographic Change research group -OPIK, University of the Basque Country, Leioa, ES; 3 Fundación Gaspar Casal and Deusto Business School, University of Deusto, ES; 4 Agirre Lehendakaria Center, ES; 5 Faculty of Education and Sport, University of Deusto, ES

**Keywords:** compassionate communities, participatory research, end of life, integrated care, social innovation

## Abstract

**Introduction:**

In the face of a growing ageing population and rising care needs, compassionate communities seek to visualize the community as an equal partner in the complex task of providing quality social and health care at the end of life.

**Description:**

Getxo Zurekin is a social innovation example for the creation of a compassionate community in Getxo, one of the most populated cities in the province of Biscay, with 25.46% of its population aged over 65. Mixed methodologies have been applied, active listening and co-creation of actions and strategies towards improving care and quality of life for people and families facing advanced disease and end of life situations, with more than 80 people interviewed to conform the basis for a collective sense making. The initiative has reached more than 1,000 people in Getxo.

**Discussion:**

Following a systemic approach, horizontal relationships and cross-sectoral collaborations have allowed engaging the active involvement of local agents in the collective sense making and co- creation process.

**Conclusion:**

Getxo Zurekin represents an example of a participatory action research model, which has shown to be effective to meet initial targets towards creation of a compassionate community.

## Introduction

People living with a life limiting illness commonly encounter anxiety, depression, social isolation, social stigma, social rejection, family breakdown, premature job loss, financial strain, spiritual dilemmas or crises, even suicide, among a host of other troubles, which are key determinants of quality of life for the dying, their carers and the bereaved [1]. Health services and palliative care services particularly, struggle to address all these social and psychological issues in an effective and timely way, while undermining the importance of community efforts to provide support and care for death, dying, loss and practical caring.

Hence, a broader approach of disease, dependency and end of life requires the involvement, engagement and empowerment of all community stakeholders –from patients, carers and citizens to care providers, including academia, NGOs and policymakers. A commitment to developing new public health models with an integrated care approach that include communities is more necessary than ever. In this sense, although transformational integrated healthcare propositions have been put forward in the Basque Country [2], evaluations have noticed the difficulties and scarce level of development in the field of community health that has been achieved since its rollout [3].

In addition, the upcoming phenomenon of the increasing number of ageing people, along with its epidemiologic trend – the increase in chronic and degenerative diseases-, posed significant economic, health and social challenges into our societies nowadays [4], and represents an expected increase in the number of people and families with long term care and dependency needs.

Compassionate communities are derived from the WHO concept of ‘Healthy Cities’ or ‘Healthy Communities’, after bringing health promotion and public health ideas to palliative care, understanding that promotion of end of life care, dying and bereavement are everyone’s responsibility [5]. In 2013, the Public Health Palliative Care International [6] was launched as an association to promote the philosophy, values, concepts, and methods of health promotion into palliative care services everywhere. This approach is the one that views the community as an equal partner in the long and complex task of providing quality health care at the end of life [7]. Since its inception, these community engagement initiatives have spread internationally, and in the last years, several organizations from different countries have boosted the development of compassionate communities with different models, but one common vision [8].

Previous experiences have shown that implementation of these kinds of initiatives are highly context-dependent and that their success requires a deep understanding of the dynamics and networks of care, just like the needs and aspirations of patients and caregivers at the end of life. In addition, it is noteworthy considering the complex reality of integrated care design and implementation, and thus using complexity theory and associated methodologies to best tailor this type of initiatives within specific contexts [9].

In this sense, the initiative of Getxo Zurekin presents a social innovation example that seeks to create a listening platform to capture communities’ narratives, while empowering and involving them in a co-creation process. The case of Getxo Zurekin presents an example of community-centred, multi-sectoral approach in the creation process of a compassionate community in the Basque Country.

This case study aims to provide readers with a descriptive analysis of the compassionate community development in the city of Getxo, while highlighting its community based participatory research principles, as well as its first observed impact and lessons learned. This work seeks to contribute with a clearer perspective on the contextual factors and the implementation of demonstrated inclusive practices to the existing integrated care research [9].

## Description of the initiative

### Overview

Getxo Zurekin is a social awareness, training, research, and care network to accompany people and families facing advanced chronic disease, end of life situations, bereavement and the solitude one faces as a consequence. The initiative was launched in 2017 with the aim of bringing into power healthcare and social care professionals and the community, as leaders in the compassionate community movement.

With more than the 25% of its population aged over 65, Getxo is one of the most aged cities in the province of Biscay [10]. So from its origins Getxo Zurekin was an opportunity to create a new integrated palliative care model in the city, as a means of improving the quality of life of citizens and contribute to the sustainability of health systems in face of growing care demands [11].

Therefore, in the Getxo Zurekin initiative, social innovation approaches were adopted to tackle the complexity whilst promoting suitable and transformative community action. The initiative seeks from its beginning to: 1) understand how the community answers the needs of those in need of palliative care and get relevant insight into the perceptions and improvement opportunities; 2) identify an underlying narrative of change that is aligned with the sociocultural characteristics of the community; 3) make communities become more involved in comprehensive palliative and end of life care; and 4) gain understanding of social networks in terms of social capital and community development theories in the context of end-of-life caring.

The adoption of the social innovation focus consisted of mixed methods that were applied for active listening and co-creation of actions and strategies towards improving care and quality of life for people and families facing advanced disease and end of life situations. Leading edge practices in social innovation, active listening tools, shared leadership, and a wide range of initiatives, without apparent cohesion but in strong connection, take place [12].

Community listening platforms are effective strategies for making the most of the collective intelligence and enable the emergence of new local adapted practices. These types of action research approaches seek transformative change through the simultaneous process of taking action and doing research. It enables working with community stakeholders to create more equitable research processes and at the same time achieving social change [13]. Using co-creation, implementation, and shared evaluation approaches are found to be better solutions, in comparison with timely and isolated consultations to experts even considering citizen queries [12]. The real community development enables communities to utilize available support systems, problem solving, decision making, and communicate and act more effectively [5]. In addition, it has been shown how sustainable innovation projects build on constant experimentation spaces and non-linear frameworks, and how they require active community listening mechanisms that guarantee continuous adaptation to best adapt to local context while generating systemic impacts in the long run [12].

### Innovative methodologies

The adoption of a social innovation approach in Getxo Zurekin focuses on building an Open Innovation Platform (OIP) to connect community agents and initiatives with the same vision to improve quality of life of local people and families facing dependency, advanced illness and end of life situations in terms of integrated palliative care. The aim is to establish common objectives, working methodologies and evaluation criteria to create synergies and gain visibility of all of them, instead of drawing on isolated actions and projects. The OIP recognizes the unique strengths that each community partner brings to the changing process.

**Figure 1:**
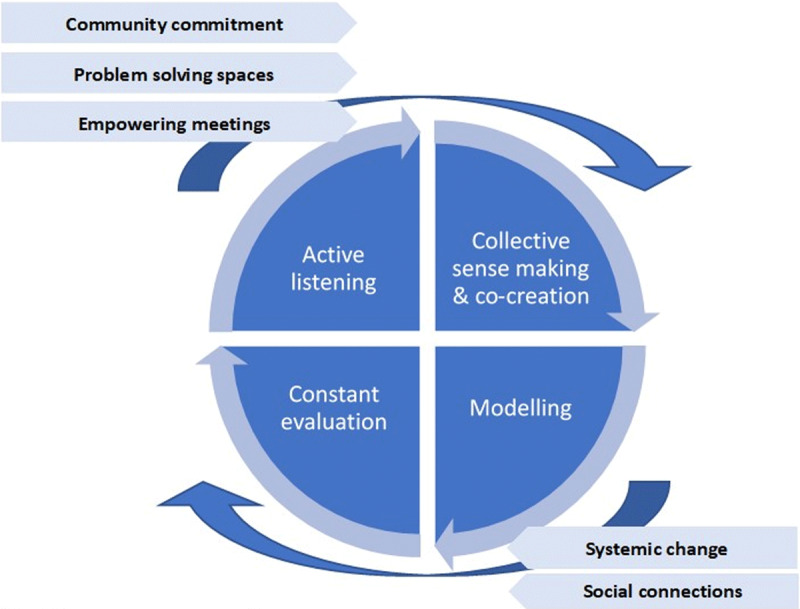
Social innovation process. Source: Elaborated by authors.

Four iterative, non-linear phases have been adopted to develop the project, based on the social innovation spiral concept [14]:

- listening – to know the perceptions and to obtain the in-depth knowledge of the real challenges and needs of people with advanced illness and end of life situations-;
- co-creation- a sense- and meaning-making local process to generate solutions and ideas built on a common narrative for change-;
- modelling – piloting initiatives in the local context derived from the discussion and empowering meetings; and
- evaluation- continuous communication and assessment of initiated actions to detect improvement areas, while adapting to emergent new narratives from the community and increasing the scope of participation and people’s empowerment (***Figure 1***).

#### Listening

A mix of methodologies and spaces have been employed as a means for identifying in depth the challenges that face people in the community in relation to advanced disease situations, end of life and dying, as well as the assets and circumstances- opportunities and barriers-, that operate in the local context for the design and implementation of innovative actions from a communitarian perspective.

**Figure 2:**
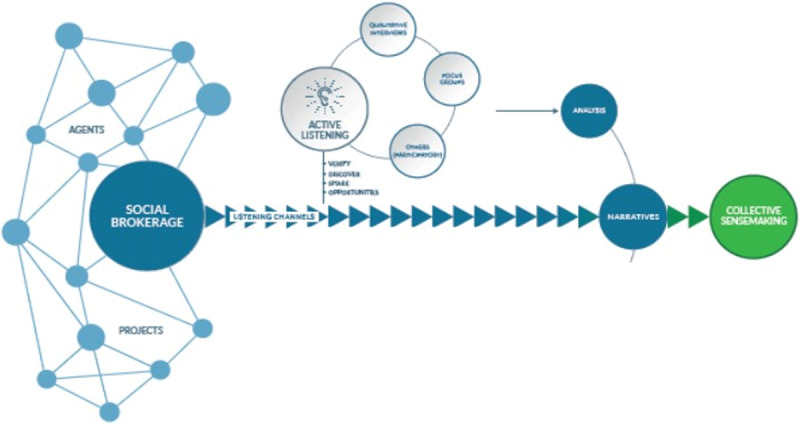
Iterative listening and co-creation phases for collective sense making. Source: Adapted from Agirre Lehendakaria Center for social and political studies.

As part of an ethnographic action research, this phase consisted of going deep into the real perceptions and perceived challenges of the different agents, operating in the community –from patients and families to health and social professionals, public services, local administration and policy makers, as well as local business and third sector partners- (***Figure 2***).

Formal in-depth semi structure interviews were held between October 2017 and November 2020, in addition to organizing Death Cafés, awareness workshops and informative talks. All interviews have a double objective: to listen and build a shared narrative in Getxo with respect to how people live end of life situations and advanced illness, and to contribute to the social awareness and involvement of people in the initiative.

***Table 1*** presents a synthesis of the ideas and examples of quotations from the interviews based on what each of the different agents and profiles mentioned.

**Table 1:** Synthesis of main ideas from interviews: challenges and opportunities.


PROFILE/ROLE	MAIN IDEAS AND RELATED QUOTATIONS

Government and social services managers	**Lack of services for the demand with an increase of care needs for these people and families** “The social services cannot cope with all the demands that need to be met. It is important that we recognize our weakness something that we find very difficult”. “The management of the social services is very complicated. It is not integrated and is not flexible”. “We are not taking into account the demographic changes, it is becoming increasingly difficult for us to identify the people”.

Health services professionals	**Basque Health Services does not reach and cannot attend all needs of everyone in end-of-life situations** “(…) The system helps me with chemo and surgery, but not with the other part – that is what I miss”. “There is an area of mental health care, but it is not enough for what we need”. **Challenges for attending situations of end-of-life and dependency with home-care services** “They are trying to tell us that looking after people at home is cheaper but that is not true. The reality is that it is way more expensive”. **Health services professionals lack specific training to care and support people in end-of-life situations** “There is no training for health care professionals to better care for people and their families in end of life”. “The medical reports do not represent the people’s reality at all, the person is dying and we know nothing about their social situation, we only know if they are getting worse o if they die without giving any detail to what happened, the process…”. **Palliative care, end-of-life situations, and bereavement are not a priority for managers and governments** “I would love to take some time out to be able to train better”. “Anything to do with education and free time seem to be the priority, however anything that has to do with bereavement, death, suffering is not a priority …”. **Change should start from fiscal policy changes** “Everyone knows that in order to change the system we must build a more balanced tax system”. “We have to start looking at out healthcare system in a different way. We need to see it as part of a system”. **Social and health services quality varies according to the professional that looks after you** “We need someone who can detect vulnerability. A professional who is trained and is flexible and has an integrated vision of how it should be. “ **Economic inequalities have an impact on the quality of care you benefit from, public services do not cover all needs** “With money everything is possible. I can get a lawyer to fight to show my level of dependency”. “(…) If you don’t have money, it is all too expensive, it can cause you to be completely broke”. **Health and social services interaction are not dynamic** “I have colleagues who will tell you that social needs are not their responsibility”. “The health sector and the social sector are completely separate, this is a complete disaster”.

Educational professionals	**Teachers are not prepared for dealing with end-of-life situations or bereavement in schools** “At the moment there is a level of sensitivity but it is not enough, we need more training”. “We are educating our children in a society that we only want to be happy; they have to be happy….but, life is not only that”. **Professionals lack from tools and abilities to face these situations** “People die often and yet we are not ready, we have to call a specialist when it happens, we don’t know what to do”. **Not dealing correctly with end-of life situations may advocate children to academic failure at school** “If you cannot deal with loss in life it is very difficult to move forward”. “(…)I was a failure at school because I had not been able to work through the loss of my mother”.

Culture and other community members	**Support from social services is usually functional and supportive in their treatment** “When you talk to someone by phone and just listen to them for half an hour you can see how their anxiety lowers. They feel less alone. But this happens once a month at the most. What do they do the rest of the time?”. **People do not know what services exist and are available** “People do not know who does what and where to call”. **Administration and formal care services structures do not facilitate the promotion of compassionate communities** “I have felt really bad. But I am not as badly off as others because in the end I am ok. I have a bit of get up and go still left in me, I still want to live life. But I have felt a bit left out people don’t know what to do with me, what to say….”. “Sometimes people call us because they feel we are closer to them and then the social services get annoyed and don’t understand why people don’t call them. But in the end of the day people see the social services as an agency to look for services both financially and logistics not so much for emotional needs”. **Effective care and support need to be adequately resourced** “To put into place community initiatives we need resources otherwise we have created an amazing network but there are no resources to do things”. **Social care structures do not take advantage of the people in the community** “The system of social protection treats the elderly like children. They are many retired people with free time, health and the desire to contribute and do things”. **People tend to feel loss after dedicating years to care from their family members** “There is a group of women who are 60+ and have spent all their lives caring for others, their families, their parents, and their children. When their children grow up they are at a loss they don’t know what to do with themselves and they become very isolated”. **People in these situations used to be stigmatized** “People are afraid to ask for help”. “We live in a society that gives when it feels it will get something back”. **Human capital remains in associations, which compete sometimes amongst themselves** “(…) in the associations and community, grassroots movements, there is competition between themselves. When a new movement is created the sensation is that here comes another that has to get part of the Kitty”. “Some associations have more privileges than others and no one seems to know why”.


Source: Prepared by authors.

Training courses have also allowed approaching reality, while providing informal carers and community with tools and capabilities to better address of people’s needs in situations of frailty, dependency, end of life or bereavement.

Active listening channels are designed and kept continuously open and active to generate data and capture changes for continuous evolvement of social innovation actions and initiatives for improving palliative care and advanced disease situations in Getxo.

#### Co-creation

Throughout the listening activities, co-creation sessions were led to propose new ideas and services that respond sustainably to the demands in the community. Heterogeneous groups of people were brought together with a double objective: first, to reconfirm the narratives arising from people in the community, and agreeing to a common interpretation of the reality; and second, to invite people to brainstorm and put ideas forward for further development of actions and solutions for meeting local peoples’ needs with respect to frailty, dependency and end of life situations (***Figure 2***).

Co-creation sessions were held both face-to-face and online, led by the development group partners who follow a sense making approach [15] to encourage the discussion and allow the collection of operating narratives common to all. Representatives of public administration, private organizations, third sector entities, academia and civil society took part.

Collective creation sessions have led to the identification of initiatives to reinforce compassionate environments in Getxo and suppose a first step and opportunity to experiment possible actions that afterwards could be put in place in different settings to promote compassionate communities.

These are some of the initiatives that have arisen from the co-creation sessions and have been initiated with priority in Getxo:

- **Death Café sessions**, where participants have coffee while they speak about illness, end-of-life and death.
- **Community influencers:** *“Activa tu comunidad”* project, which aims to promote a mutual support system within neighbourhoods for people in situation of dependency, frailty and advanced illness situations with care needs and those bereaved.
- **Bereavement groups:** led by members of the community who have themselves lost a loved one, being accompanied by a professional grief counsellor.
- **Training programs** for people providing formal care; for example, migrant women.
- ***“Lokarria”* project**, which is based in the creation of a community figure that aims to promote social cohesion and serve as a linker between community and public administration services.
- **Community Theatre:** in collaboration with a local community, we bring to life through theatre the narrative around death and dying in this community.

#### Modelling

The identification of new ideas that would meet community needs were followed by their collaborative development. Implementation started at small and medium scale for testing, with subsequent scaling up. This prototyping approach represents a sharp departure from traditional planning-based responses to complex challenges [16].

A variety of activities have been deployed in the city of Getxo targeted to different community members, from mutual support groups to Death Café meetings, as well as specific training programs. A wide portfolio of actions diversifies the risk when promoting transformational change, while favouring change at multiple levels and fields of action.

#### Evaluation

Evaluation mechanisms and channels were put in place to continuously monitor activities, as well as new arising narratives that may require adaptation and changes. Organization of awareness actions, public events or training sessions offer spaces for listening and involving citizens and community actors in the initiative. This step includes the idea of maintaining a constant public narrative that guarantees keeping the project alive.

Addressing social challenges that were identified in the listening phase, while attempting systemic changes, need operating at multiple levels to respond to social needs and local challenges. Multiple groups of people need to be involved in the process, which should reflect the social diversity and bring into the project different voices, as opposed to a homogeneous group and one unique and limited perspective of the reality in Getxo (e.g. only with the presence of scientists, academics, civil servants, etc.) [16]. In Getxo, the participation of every community agent was recognized as core for its success and sustainability. Academia partners and researchers adopted a key leadership role for the adoption of social innovation methodologies and its commissioning.

## Impact

**Figure 3:**
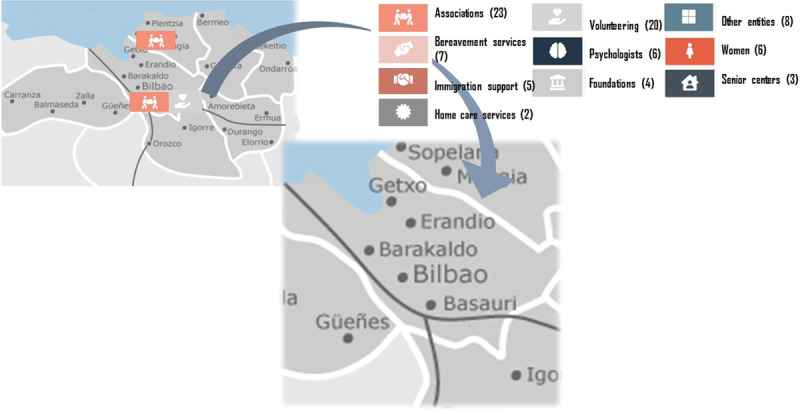
Community and institutional agents chart in Getxo Zurekin. Source: Elaborated by authors from the compassionate chart at fundacionsonrisa.com/.

The adopted social innovation approach has allowed the attachment and a broad alliance of multi-level community and institutional agents (local administration, provincial councils, third sector partners, foundations, local businesses, and citizens) in the process (***Figure 3***), both located at local and province levels. ***Figure 3*** shows an extended map of Getxo, and some of the entities that are involved in the program in the province of Biscay. Leadership from social innovation expert researchers and the commitment for achieving the involvement of every community partner in the process has allowed achieving critical mass, and at the same time explicit political will to boost the initiative.

In the initial listening steps, and with respect to the in-depth interviews, more than 80 people from the community of Getxo were interviewed. The interview outline has been previously designed and validated by an expert group in the field of sociology, political science, and ethnographic research methodologies. Likewise, questions were posed in an open form, admitting adaptation to better adjust to the specific interviewees. People participating in the interviews correspond to representatives of the public administrations (from local to regional level), professionals from public social and health organization, and community and third sector partners.

Analysis and data extraction followed a standard template through which challenges, opportunities, barriers, and facilitators were identified. This process allowed the segmentation of profiles based on the narratives and patterns of behaviours towards advanced disease and end of life care, and provide insight regarding different ways to shape local reality (***Table 2***).

**Table 2:** Breakdown of profiles included in in-depth interviews. Source: Elaborated by authors.


	N (% OF TOTAL)

**Sex**	

Woman	47

Man	23

**Age (variability)**	16-90 years old

**Profession/role**	

Primary care, palliative, HaH physician	7 (9.85)

Nurse	4 (5.63)

Psychologist	2 (2.81)

Social worker	1 (1.40)

Teaching professional	4 (5.63)

Immigrant career	6 (8.45)

Local business person	10 (14.08)

Sick people	4 (5.63)

Public administration representative	2 (2.81)

Volunteer	18 (25.35)

Informal career	6 (8.45)

Funeral service/cemetery professionals	3 (4.22)

Religious	1 (1.40)

Student	1 (1.40)

Journalist (with loss history)	2 (2.81)

HaH: Hospitalization at Home	


Comprehensive interviews allowed to gather more than 90 narratives, since some respondents brought more than one narrative as they experienced end of life situations from different walks of life and brought their narrative to the project from different social positions and life experiences (an example represents the health professional that was also going through a mourning process). These formed the basis for the collective sense making and subsequent co-creation sessions.

Along with other awareness and training activities, since its beginning in 2017, the initiative has reached more than 1,000 people in the city of Getxo and more than 2,000 people through external dissemination activities in international congresses.

***Table 3*** presents the breakdown of training and sensitization activities, in addition to the number of people reached in its case and year.

**Table 3:** Listening activities and outreach. 2017–2021.


YEAR	TYPE OF ACTIVITY	NUMBER OF ACTIVITIES DONE	NUMBER OF PEOPLE REACHED

2017	Talks/Workshops	3	145

	Death Cafés	2	30

	Training courses	1	20

	Conferences	2	1,100

2018	Talks/Workshops	23	847

	Death Cafés	12	199

	Training courses	6	230

	Conferences	6	2.680

2019	Talks/Workshops	6	250

	Death Cafés	10	94

	Training courses	11	207

	Conferences	2	1,240

2020	Talks/Workshops	8	200

	Death Cafés	5	64

	Training courses	4	40

	Virtual seminars	2	120

	Individual telephone support (COVID derived)		18 families with care responsibilities, in charge of dependant members or oncology processes.


Source: Prepared by authors.

## Discussion

Getxo Zurekin is part of a global movement to change attitudes and behaviours around death, dying and bereavement. As other experiences around the globe, it seeks to reinforce the recognition of care for one another, and the idea that loss and bereavement is not simply a task solely for health and social services but is everyone’s responsibility [1,17,18,19]. Experiences were developed in different municipalities of the Basque Country using a similar approach to create cities that recognize the responsibility of collective caring in end-of-life situations. Cities such as Santurtzi, Vitoria-Gasteiz, Zarautz, Orio, Bidasoa, Tolosa, Elgoibar and Donostia represent some of the places that formed the network of Compassionate Communities in the Basque Country [20].

The description of the Getxo case intends to fill a gap in the existing literature and provide a direction and framework to guide the development of similar projects in other cities.

A growing interest and expansion of compassionate community experiences has taken place in the past number of years. Although the same philosophy and vision is shared by all experiences, different approaches and models have been adopted to their realization (8). In contrast to top-down approaches, where public organizations and governments put in place policies to offer more integrated, compassionate, and sustainable care models [21,22,23], Getxo Zurekin comprises an example of a community participation model, where identification and demands for a more integrated and sustainable model of care comes up through collective and community sense making. Furthermore, co-creation and development of actions rely on the close involvement and constant participation of community members.

Amongst the key elements identified in the experience of Getxo, the development of networks between professionals, community providers and researchers contributed to the progress of the initiative. Providing spaces to share experiences, needs and different views in the field of advanced disease and end of life care, supported the creation of social networks around these people. Previous research has also reported these elements to be valuable for the creation of compassionate communities [24,25]. In line with several authors [24,26], the experience of Getxo Zurekin has put in place social awareness actions, or programs for training caregivers, as key elements for the development of a compassionate community in the city of Getxo.

One of the meaningful differences in the Getxo Zurekin experience is built upon the idea of collecting information and drawing on the narratives of people living in the community, beyond the quantitative and qualitative data coming from the local public institutions and other sources. The underlying assumption of this idea is that considering different sensitivities and perceptions, which in the end will affect public policies and measures undertaken in real context, is a core element for transformative systemic change. The collective and participatory interpretation of the various narratives is also embedded in the strategy towards mobilizing people on the issue of compassion and building a series of actions for responding to the existing needs.

With respect to specific initiatives put in place, the development of a “community promoter” in Getxo, named as “lokarria” in Basque, has also been proposed and implemented with success in other settings [27]. Its prototyping and first implementation were scarcely monitored, and it exceeds the scope of this paper.

Similarly, training and awareness activities, death cafés and neighbourhood networks were also developed in Getxo, just like in other compassionate community models described by several authors [8].

Although Getxo Zurekin was initially conceptualized as a learning experience with a local approach to facilitate community co-design of actions and strategies towards the development of an integrated palliative care model, where health, social, and community sectors interact, it has the ambition to transcend its potential of social transformation globally. In the last year, the initiative is collaborating with local institutions and civic organizations to share lessons learned with other municipalities in the region of the Basque Country, and currently, there is an ambition to transcend local boundaries while extrapolating best practices and lessons learned to a regional level.

Further research should focus on the evaluation of the satisfaction of local people and caregivers with the process, the enhancement of third sector associations, patients, carers, and citizens’ empowerment, social support, as well as the impact on patients and carers’ wellbeing and health outcomes. Other research lines could also contribute showing the potential economic savings, resulting from community case management interventions in formal care for public health and social services in Getxo, as it has been done in for other settings [21,28].

## Lessons learned

- The participatory approach and working methodology support a collective and shared analysis of the local reality and encourages development of further lines of work.
- The success of a participatory process depends on offering spaces for the participation and consideration of different sensitizations, rather than attending to the volume and mobilizing bigger groups of people.
- Promoting horizontal relationships between local administrations and community is as essential as gathering qualitative data.
- Projects with a transformational nature such as Getxo Zurekin require a systemic approach and development of actions at different levels (community engagement, start- ups, public-private collaborations, public services, and regulation). Ensuring a critical mass from a multilevel perspective may support the development of actions at different levels and fields, which is key for the implementation of systemic changes.
- Adoption of social innovation methodologies have shown to be effective in achieving objectives in a change demanding context in health.

## Conclusion

Getxo Zurekin is a participatory social innovation initiative, which has proven to be effective in achieving its targets in the city of Getxo. It presents an example of how to apply participatory approaches and social transformation methodologies within a community. Its development shows several lessons learned to be considered in other settings. Further research should focus on the evaluation of the pilot initiatives as well as satisfaction of community members with the process and results.
